# Relationship between cognitive functioning and physical fitness in regard to age and sex

**DOI:** 10.1186/s12887-023-04028-8

**Published:** 2023-04-29

**Authors:** Francisco Tomás González-Fernández, Gabriel Delgado-García, Jesús Siquier Coll, Ana Filipa Silva, Hadi Nobari, Filipe Manuel Clemente

**Affiliations:** 1 Department of Physical Education and Sports, Faculty of Sport Sciences, University of Granada, Granada, Granada, 18071 Spain; 2 SER Research Group, Department of Physical Activity and Sport Sciences, Center of Higher Education Alberta Giménez (Affiliated to Pontifical, University of Comillas), Palma, 07013 Spain; 3 The Research Centre in Sports Sciences, Health Sciences and Human Development (CIDESD), Vila Real, 5001-801 Portugal; 4 Escola Superior Desporto e Lazer, Instituto Politécnico de Viana do Castelo, Rua Escola Industrial e Comercial de Nun’Álvares, Viana do Castelo, 4900-347 Portugal; 5 Instituto de Telecomunicações, Delegação da Covilhã, Lisboa, 1049-001 Portugal; 6 Department of Exercise Physiology, Faculty of Educational Sciences and Psychology, University of Mohaghegh Ardabili, 56199-11367 Ardabil, Iran; 7 Faculty of Sport Science, University of Extremadura, Cáceres, Spain

**Keywords:** D2 attention test, Attention, Concentration, Physical fitness, Cognitive, Sports activity

## Abstract

The aim of this study was to analyze the relationships among physical cognitive ability, academic performance, and physical fitness regarding age and sex in a group of 187 students (53.48% male, 46.52% female) from one town of Norwest of Jaén, Andalusia (Spain), aged between 9 and 15 years old (*M* = 11.97, *SD* = 1.99). The D2 attention test was used in order to analyze selective attention and concentration. Physical fitness, reflected on maximal oxygen uptake (VO_2max_), was evaluated using the 6 min Walking Test (6MWT). The analysis taken indicated a significant relationship between physical fitness level, attention, and concentration, as in the general sample looking at sex (finding differences between boys and girls in some DA score in almost all age categories [p < 0.05]) and at age category (finding some differences between the younger age category groups and the older age category groups in some DA scores (p < 0.05), not finding any significant interaction between sex and age category (p > 0.05). In sum, the present study revealed that students with better aerobic fitness can present better-processed elements and smaller omission errors. Moreover, girls and older students seem to present better cognitive functioning scores than boys and younger. Our findings suggest that more research is necessary to elucidate the cognitive function between ages, sexes, and physical fitness and anthropometry levels of students.

## Introduction

Current sedentary behavior of children over the last decade has created a huge problem for society. The development of video games and mobile screens has led to children spending more time playing video games to the detriment of physical activity [1]. Sedentary lifestyles in children trigger metabolic diseases such as obesity [2] or other psychological illnesses such as depression [3]. Levels of physical activity (PA) are reported to be lower than in previous years and continue to decline [4], exacerbated by the pandemic situation [5]. Siquier Coll et al. [6] mentioned that there is a high number of children and pre-adolescents who do not achieve the recommended level of PA. This study found lower cardiovascular values and body composition in sedentary children compared to groups of children practicing in different sports. Another research reported that higher sedentary hours were associated with lower cardiorespiratory fitness [7]. Besides, physical inactivity has been reported to be related to poorer attention and academic performance of students in primary and secondary schools [8].

Elsewhere, it has been suggested that aerobic capacity is the best predictor of cognitive function [9]. Reigal et al. [10] stated that oxygen consumption is the best predictor of attentional parameters. Similarly, a recent systematic review reported that higher aerobic capacity is related to higher academic performance [11]. Therefore, research on physical activity levels, academic performance and attention has been booming in recent years [12–14]. PA involves the development of attention in childhood [15], especially in open-task sports [16]. These sports require decision-making in which the child needs to be highly attentive. Recent study reported improvements in selective attention in children with attention deficit hyperactivity disorder [17]. Nevertheless, benefits in attention in closed-task PA have also been reported. Herold, Behrendt, Meißner, Müller, & Schega [18] found enhancements in the d-2 attention test acutely following sprint interval training. Another recent study reported an association between the D2 attention test and fitness level [10].

Likewise, Chen, Gu, Chen, & Wang (2022) [19] found an association with D2 attention test and cardiorespiratory fitness in schoolchildren. Hogan et al., [20] reported that physical fitness and acute exercise could improve cognition by increasing the functionality of the attentional system in adolescence. Attention, an aspect of cognition, has also been reported to be closely linked to aerobic fitness throughout the human lifespan [21, 22]. Furthermore, PA improves attention through increased release of transmitters involved in cognitive processes [22]. Thus, low fitness levels appear to be correlated with poorer cognitive skills [23]. Higher fitness levels are associated with higher executive functioning performance in different conditions of the Erikson flanker task, and also with higher P3 amplitudes of the event-related potential (ERP) in response to stimuli. This fact suggests that a stronger allocation of attentional resources during stimulus encoding is related to better performance in more physically fit children [20]. Possible physiological mechanisms of exercise-induced neural adaptation are the promotion of cerebral vascularisation, up-regulation of genes associated with cellular plasticity, increased regional blood flow, and increased levels of brain-derived neurotrophic factor (BDNF) [20]. Hence, it is necessary to study the relationship between cognition and physical activity levels. The relationship between attention and physical activity levels has been established [15]. However, few studies have assessed the relationship between cardiorespiratory fitness and attention in different age groups and sex differences [9]. Therefore, there is a need for this study, which could lead to a better understanding of the topic. Based on the above, it is hypothesized that the students with higher values of physical fitness, concretely in maximal oxygen uptake (VO_2max_) will have better performance in cognition, reflexed in different values of D2 attention test. Thus, this study aimed to observe the possible relationships between aerobic fitness and cognition in a specific test (d-2 test).

## Materials and methods

### Study Design

This study followed a cross-sectional design. Convenience sampling was performed. The study was conducted in January 2022 and students were assessed three times. The first, second, and third assessments were separated by one week. The purpose was to understand the relationship between the cognitive ability reflexed in the D2 attention test, and physical fitness through 6 min walking test considering the sex and age as moderators. During the assessment, 12 participants were excluded [inability to perform the D2 attention test (n = 6) and only performed one or two of assessing (n = 6)]. Finally, 187 participants remained and completed the entire experiment.

### Participants

A total of 187 students (53.48% male, 46.52% female) from two schools from the region of Andalucía, participated in the present research. Students were recruited from one village of the province of Jaén with a population ranging from 5,000 to 10,000 inhabitants according to the National Institute of Statistics from the Spanish Government (http://www.ine.es/; accessed on 12 February 2022). A priori sample size calculation was performed using a free on-line tool, G*Power (www.gpower.hhu.de), with a power level of 95% and an α level of 0.05 and based in previous and similar studies [10], revealed that the sample size of > 160 would be sufficient for the analysis.

#### Informed consent

s were obtained from parents of all the participants and signed regarding all participants involved in this study. The study was conducted according to the Declaration of Helsinki and was approved by the ethics committee of institute’s research of the Pontifical University of Comillas (2021/89).

On the one hand, inclusion criteria were: i) students that were assessment during all experimental sessions. On the other hand, exclusion criteria for participants in this study were (i) reported normal vision and no history of any neuropsychological impairments that could affect the results of the experiment and (ii) have a health problem that could bias any result or prevent you from taking a test of the study, and (c) not have parental authorization.

## Measures and Instruments

### Anthropometry

Height and body weight were collected at the beginning of assessment, at the same hour and at the same day of the week (between 9:30 a.m. to 11:30 a.m.). Height was measured using a stadiometer (SECA 213, Birmingham, UK) to the nearest 0.1 cm, and players were asked to remove their shoes and other accessories that could influence the assessment. Students also had to be in a vertical and immobile position, with arms extended along the body, and look straight ahead in an upright position. For each measure, only one measurement was collected.

### Physical fitness

The 6 min Walking Test (6MWT) is a sub-maximal exercise test used to assess aerobic capacity and endurance. It was developed by the American Thoracic Society, and it involves a corridor with a length of at least 30 m. The length of the route should be marked every 3 m, the return points should be marked with cones and the starting point should be indicated with a brightly colored tape. All participants were reported to walk back and forth in a straight line as fast as possible with self-paced over a 6-minute period of time. During the 6MWT, all the subjects were informed every 1 min. HR was measured during the 6MWT by telemetry (Heart Rate Transmitter Model T34; Polar). Once 6MWT is over, the total carried out distance over 6 min was registered as the result of 6MWD test. To confirm the reproducibility of the 6MWT, 80 boys randomly were asked to repeat the 6MWT with an interval of 1 week [24, 25].

### D2 attention test

Selective attention was measured with the D2 attention test individually (Brickenkamp & Zillmer, 1998) [26]. It is used to analyses the visually ability to scan and select certain relevant aspects of a task, while ignoring irrelevant ones quickly and accurately. It consists of 14 rows of 47 characters each, with a total of 658 elements. The items can be “p” or “d” with one, two, three, or four dashes, arranged either individually or in pairs at the top and/or bottom of each letter. Thus, the “d’s” with two dashes - regardless of position- should be crossed. Subjects have 20 s per row and have to mark as many of those d’s as possible. The scores that subjects can obtained were the following: TR (processed elements), TA (successes), O (omissions), C (commissions or errors), TOT [effectiveness in the task = TR− (O + C)], CON (concentration = TA − C), TR+ (last stimulus analyzed in the row with the most attempted elements), TR− (last stimulus analyzed in the row with the least attempted elements) and VAR [index of variation between the last stimulus analyzed between different rows = (TR+) − (TR−)]. This test has a test–retest reliability in the original study up to 0.90.

### Procedure

Sample set was obtained from two schools. Every school management was contacted in order to request them to participate. In addition, written and informed consent was obtained from the parent/guardian of each adolescent who wanted to participate. Evaluations were taken along 24 to 72 h difference between them.

Physical fitness (6MWT) was evaluated on the first day during the class of physical education, attention and concentration were evaluated in the second day and finally, academic performance was recollected by the advisor of both schools since academic bulletin (always with the authorization of responsible). The evaluation schedules were from 9:00 to 13:30 in the morning.

The D2 attention test was taken following the protocol established in the test. Instructions were carefully explained to the students and previous questions were solved in order to avoid any doubts. In all cases, the explanation was performed by one education phycologist. The test was conducted in groups in a classroom at the school. For the physical tests, a 10-min warm-up was carried out. Activation and joint mobility were developed. Subsequently, the 6 min walking test was conducted.

### Statistical analysis

Data analysis was performed using Real Statistic Using Excel software. Data are mean ± standard deviation, unless otherwise stated. Significant p-value was established at p < 0.05.

A two-factor ANOVA was conducted to test for differences in the attentional variables by age category and for sex. Our focal variable was age category, and our moderator variable was sex. ANOVA was performed using regression analysis. Partial eta squared (η^2^) was used as effect size. To interpret the magnitude of the eta squares, we adopted the following criteria (Cohen, 2013): r ≤ 0.003, no effect; 0.010 ≤ r < 0.060, small effect; 0.060 ≤ r < 0.140, intermediate effect and eta square ≥ 0.14, large effect. Normal distribution and homogeneity tests (Shapiro-Wilk’s test) were conducted on all metrics and the studentized residuals based on the regression line (p > 0.05). Simple effect of age category and sex was also evaluated. Tukey HSD for two factor ANOVA was also used for post-hoc testing (for age category and sex). In addition, a Pearson’s correlation coefficient r was used to examine the relationship between age category and different DA scores, VO_2max_ and Body Mass Index (BMI). To interpret the magnitude of these correlations, we adopted the following criteria: r ≤ 0.1, trivial; 0.1 < r ≤ 0.3, small; 0.3 < r ≤ 0.5, moderate; 0.5 < r ≤ 0.7, large; 0.7 < r ≤ 0.9, very large; and r > 0.9, almost perfect. The effect size was assessed following Cohen’s scale (2013): (I) 0-0.20, “negligible effect”; (II) 0.20–0.50, “small effect”; (III) 0.50–0.80, “medium effect”; (IV) 0.80-1, “large effect”.

## Results

Descriptive statistics were calculated for each variable (Table 1).

**Table 1:** Participants’ characteristics (Mean ± SD) of the present study (total N = 187) by age category

	Age category						
**Variable**	9 y	10 y	11 y	12 y	13 y	14 y	15 y
**N**	23	39	30	11	38	27	21
**Height (cm)**	140.7 ± 7.1	146.2 ± 12.5	149.2 ± 7.7	157.3 ± 4.9	162.0 ± 6.5	164.9 ± 7.1	164.6 ± 6.8
**BW (kg)**	38.13 ± 10.03	39.72 ± 10.14	43.01 ± 11.01	55.12 ± 12.67	54.80 ± 11.86	56.50 ± 11.18	57.40 ± 8.69
**BMI (kg/m** ^**2**^ **)**	19.15 ± 3.54	18.54 ± 3.73	19.08 ± 3.49	22.19 ± 4.52	20.76 ± 3.54	20.64 ± 2.91	21.06 ± 1.77
**V02**_**Max**_ **(ml/kg/min)**	35.43 ± 3.28	38.67 ± 5.32	38.06 ± 4.61	34.75 ± 5.45	34.04 ± 4.72	34.11 ± 3.90	34.15 ± 2.14

SD: Standard deviation; y: years; BW: Body Weight; BMI: Body Mass Index; VO2 max.: maximal oxygen consumption

The two-factor ANOVAs conducted with the different DA scores (TR score, TA score, TR + score, TR- score, O score, C Score and DA Score) showed no statistically significant interaction between sex and age in any case (p > 0.05). (See Table 2 for more information)

**Table 2:** Summary of the Two-Way ANOVAs performed, including sex and age category as independent variables and the different DA scores as dependent variables

DA score	Comparison	Two-Way ANOVA parameter				
		df	MS	F	p-value	P eta-sq
TR	Sex	1	11,066	2.32	0.130	0.013
	Age category	6	50,354	10.54	**≤ 0.0001**	0.269
	Inter	6	3062	0.64	0.697	0.022
	Within	172	4776			
	Total	185	6716			
TA	Sex	1	3187	1.48	0.225	0.009
	Age category	6	7099	3.31	**0.004**	0.103
	Inter	6	1698	0.79	0.578	0.027
	Within	172	2146			
	Total	185	2418			
O	Sex	1	703	1.08	0.300	0.006
	Age category	6	1019	1.56	0.160	0.052
	Inter	6	996	1.53	0.171	0.051
	Within	172	651			
	Total	185	684			
C	Sex	1	1	0.01	0.937	0
	Age category	6	153	1.25	0.284	0.042
	Inter	6	258	2.1	0.056	0.068
	Within	172	123			
	Total	185	130			
TR+	Sex	1	9	0.14	0.709	0.001
	Age category	6	339	5.56	**≤ 0.0001**	0.162
	Inter	6	13	0.22	0.970	0.008
	Within	172	61			
	Total	185	69			
TR-	Sex	1	8	0.23	0.632	0.001
	Age category	6	192	5.19	**≤ 0.0001**	0.153
	Inter	6	18	0.49	0.813	0.017
	Within	172	37			
	Total	185	44			

*df: Degree of freedom; MS: Mean Square; F: F ratio; P eta-sq: Partial Eta square; Inter: Interaction

Comparisons between sexes in each age category showed differences in the 11 years old group for the C score (p = 0.023, F = 5.23), in the 12 for the O score (p = 0.019, F = 5.57), in the 14 years old group for the C score (p = 0.024, F = 5.17) and in the 15 years group of age for TA score (p = 0.02, F = 5.33).

**Fig. 1:**
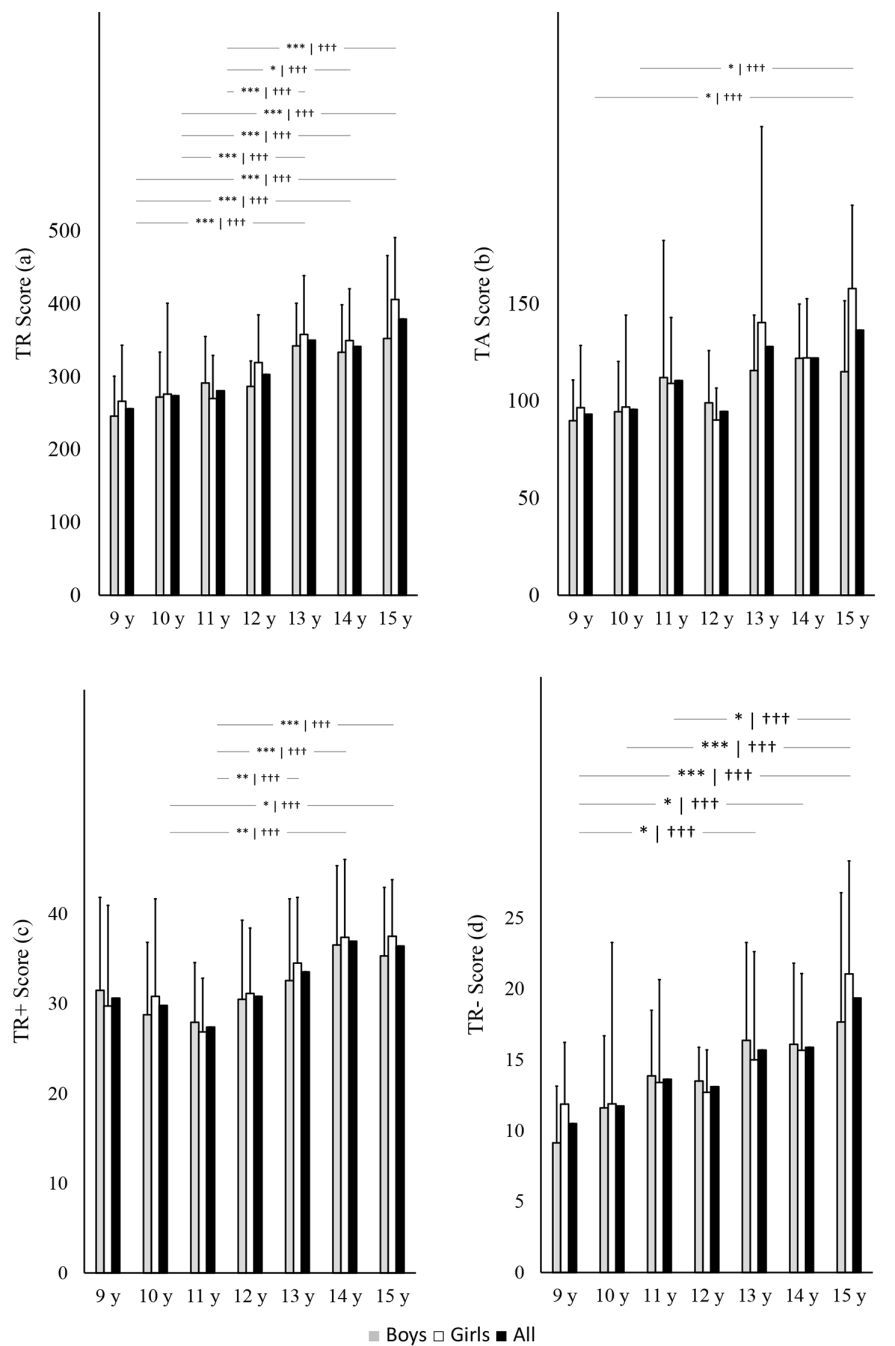
D2 test of attention scores by age category. Figure 1a refer to total responses (TR), Fig. 2b to successes (TA), Fig. 2c to last stimulus analyzed in the row with the most attempted elements (TR+), and Fig. 2d to last stimulus analyzed in the row with the least attempted elements (TR-).

Regarding comparisons among age categories, Tukey HSD showed significant differences in: (i) TR between the 9-, 10- and 11- with the 13-, 14- and 15-years old groups (Fig. 1a); (ii) in TA between the 9- and 10-years categories and the 15 years old category (Fig. 1b); (iii) in TR + between the 10 and 14- and 15-years old groups and between the 11 and 13-, 14-, 15- years old groups (Fig. 1c); and (iv) in TR- between the 9 years old group and the 13-, 14- and 15-years old group and between the 10- and 11-years old group (Fig. 1d).

As for comparisons among age categories in the case of boys, there were differences between the different categories of age for the TR (p < 0.001), TR+ (p = 0.039), TR- (p = 0.008). In the case of the girls there were differences between categories of age for TR, TA (p < 0.001), TR+ (p < 0.001), TR- (p < 0.001), O (p = 0.007) and C score (p = 0.010).

**Fig. 2:**
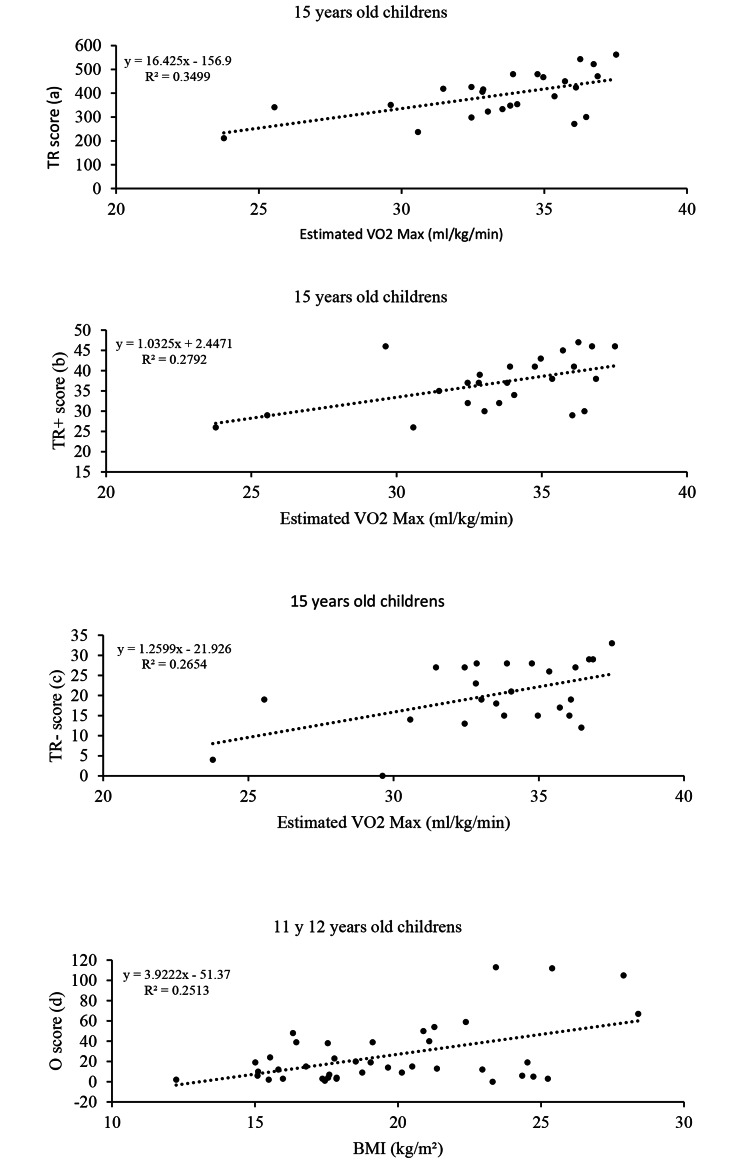
Regression lines (with large effect size) predicting the estimated VO_2max_ or the BMI, based on the DA scores

In the case of the correlations between the different DA scores and the VO_2max_ obtained in 6MWT a positive moderate correlation was found for TA in the 11–12 years old group and for O in the 9 years old group (r = 0.32, p = 0.041 and r = 0.48, p = 0.003, respectively). A negative moderate correlation was found for O in the 11–12 years old group (r = -0.43, p = 0.005). Last, a positive large correlation was found for TR, TR + and TR- in students of 15 years old (r = 0.59, p = 0.002 [Fig. 2a]; r = 0.53, p = 0.007 [Fig. 2b], r = 0.52, p = 0.008 [Fig. 2c]).

DA scores and the BMI (kg/m²) showed a negative moderate correlation with TR in the 15 years old group (r = -0.42, p = 0.037), with O in the 9- and 14-years old group (r = -0.46, p = 0.035; r = -0.42, p = 0.039, respectively) and with TR + in the 14 years old group (r = -0.44, p = 0.03). A positive large correlation was found for O in the 11–12 years old group (r = 0.50, p = 0.001 [Fig. 2d]).

## Discussion

The main aim of the present study was to analyze the relationships between aerobic fitness and cognition (in a specific test: d-2 test). As hypothesized, correlations were observed between aerobic fitness and some DA scores. For instance, in 9 years old group, a positive moderate correlation was found with omissions (O), in opposition, it was moderately negative in 11–12 years old group. A positive moderate correlation was registered with TA in 11–12 years old group. However, it was in the age of 15 years that positive large correlations were found with processed elements (TR), TR + and TR-. Moreover, higher BMI (which could mean lower PA, thus lower aerobic capacity) showed negative moderate correlations with O in the 9- and 14-years old group, with TR + in the 14 years old group and with TR in the 15 years old group. Conversely positive large correlation was found between BMI and O in the 11–12 years old group. Therefore, the present study seems suggest that a positive correlation exists between aerobic capacity and cognition (using d-2 test) in young ages.

The relationships between aerobic fitness (namely maximal oxygen uptake) and cognitive function have been presented in previous studies [26]. Additionally, students with better aerobic fitness have been also associated with better academic achievements [27]. In our study, it was found that in some ages the better aerobic fitness the TR and TR+. Additionally, the better aerobic fitness the smaller omission error. Better aerobic fitness and greater PA participation have been associated with greater P3 (endogenous component of the event-related brain potentials occurring approximately 300–800 ms after stimulus onset) amplitude [28] and faster P3 latency [29] along midline scalp sites in response to a stimulus discrimination compared to those with worse aerobic fitness. This can be one of the causes to justify the beneficial effects of aerobic fitness for improving succeeded proceeded elements and reducing the omission errors. This fact is confirmed in adults and also children and youth populations since original research showed that fitness was positively associated with neuroelectric indices of attention and working memory and response speed in children, while cognitive processing speed was independent of age [26]. Another explanation of positive effects of aerobic fitness is the possibility of greater aerobic fitness and namely aerobic exercise may increase synchrony in the firing of neural generators, with positive consequences to increase P3 amplitude and decrease P3 latency [26].

In our study, it was also found that students in 11 and 12 years old with greater BMI were the same with greater omissions. Although the BMI is not the best indicator of fat and health, overweight can be identified while using BMI. It is expectable a greater sedentary behavior and worse physical fitness in students with worse BMI may justify the linear relationship with the increased omission errors. However, these results were the oppositive in 9 and 14 years old. Possibly, the absence of markers related with fat mass can influence the results interpretation. As an example, students may present greater BMI without necessarily being related with greater body fat composition but with a greater lean mass justifying that.

Our study also revealed some differences between ages regarding cognitive functioning. Older tended to present better scores in TR, TR + and TR- than younger ones. In addition, students with 12 years old had significant greater omissions, while students with 11 years old had the greatest commissions or errors. Differences in age, namely increase in performance of attention and cognitive performance in older participants can be justified by the maturational effect. For example, younger activate the left ventrolateral prefrontal cortex rather than the expected right ventrolateral prefrontal cortex exhibited in adults [30]. The fact of the magnitude of activation of the left ventrolateral prefrontal cortex is similar to the adult’s activation of the right ventrolateral suggests that through the development, children may change patterns of activation namely recruiting different neural resources than older to achieve similar goals, albeit with less efficiency [26].

Differences in cognitive scores between boys and girls were also found. The current research revealed that girls presented significant greater values than boys in C at age 11 and 14, in O at age 12 and in TA at age 15. In opposition, no significant differences in TR + or TR- have been found. Previous studies revealed that PA interventions seem to play a greater role in benefiting girls than boys regarding performance in the D2 attention test [31]. This can be justified by the typical smaller physical activity levels of girls in comparison to boys. However, the interaction of physical fitness with the fact that girls achieve maturation earlier than boys with natural consequences in hormonal and neurological modifications may also provide a reason to girls presented better scores than boys in these sensitive ages analyzed.

The current research presents some limitations. One of the limitations is related with the absence of some contextual factors that can interact as covariables to explain some of the evidence found (e.g., academic level and achievements, other cognitive functions). Moreover, PA time and intensity per week would be beneficial to characterize the sample and also combine with information on BMI and aerobic fitness. Also, the present study had an observational character, allowing only to correlate the variables considering the different ages and genders, and not the effect that a possible improvement in the aerobic capacity would have on cognition. For future studies, we suggest evaluating this possible influence. Although those limitations, this study represents an opportunity to differentiate cognitive function between ages, sexes and physical fitness and anthropometry levels of students.

## Conclusions

The current research revealed that students with better aerobic fitness can present better-processed elements and smaller omission errors. Moreover, girls and older students seem to present better cognitive functioning scores than boys and younger. Although this evidence, the results may not be generalized since the specific relationships were not global for all ages, while the differences between sexes and ages were not always significant for all cognitive functioning scores.

## Data Availability

The datasets generated and analysed during the current study are not publicly available due to ethical restrictions, however are available from F.T.G.F on reasonable request.

## Abbreviations

BDNF: brain-derived neurotrophic factor
PA: Physical activity
6MWT: six minutes walking test. TR:processed elements
TA: successes
O: omissions
C: commissions
